# 2-Amino­pyrimidinium hydrogen oxalate monohydrate

**DOI:** 10.1107/S1600536809039907

**Published:** 2009-10-23

**Authors:** Hossein Eshtiagh-Hosseini, Zakieh Yousefi, Masoud Mirzaei

**Affiliations:** a Department of Chemistry, School of Sciences, Ferdowsi University of Mashhad, Mashhad 917791436, Iran

## Abstract

In the title hydrated salt, C_4_H_6_N_3_^+^·C_2_HO_4_^−^·H_2_O, inter­molecular N—H⋯O and O—H⋯O hydrogen bonding helps to stabilize the crystal structure.

## Related literature

For the biological properties of pyrimidines, see: Rabie *et al.* (2007 ▶). For the applications of amino­pyrimidines, see: Rospenk & Koll (2007 ▶). For amino­pyrimidine salts, see: Childs *et al.* (2007 ▶).
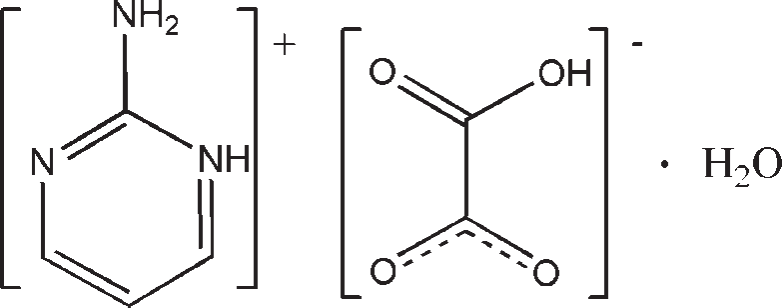


## Experimental

### 

#### Crystal data

C_4_H_6_N_3_^+^·C_2_HO_4_^−^·H_2_O
*M* *_r_* = 203.16
Triclinic, 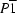

*a* = 6.295 (2) Å
*b* = 6.339 (2) Å
*c* = 11.111 (4) Å
α = 75.045 (6)°
β = 84.302 (6)°
γ = 86.026 (7)°
*V* = 425.8 (2) Å^3^
*Z* = 2
Mo *K*α radiation
μ = 0.14 mm^−1^
*T* = 120 K
0.35 × 0.15 × 0.07 mm

#### Data collection

Bruker SMART 1000 CCD area-detector diffractometer
Absorption correction: none
3983 measured reflections
1835 independent reflections
1177 reflections with *I* > 2σ(*I*)
*R*_int_ = 0.030

#### Refinement

*R*[*F* ^2^ > 2σ(*F* ^2^)] = 0.055
*wR*(*F* ^2^) = 0.148
*S* = 1.02
1835 reflections
151 parameters
H atoms treated by a mixture of independent and constrained refinement
Δρ_max_ = 0.44 e Å^−3^
Δρ_min_ = −0.29 e Å^−3^

### 

Data collection: *SMART* (Bruker, 1998 ▶); cell refinement: *SAINT-Plus* (Bruker, 1998 ▶); data reduction: *SAINT-Plus*; program(s) used to solve structure: *SHELXTL* (Sheldrick, 2008 ▶); program(s) used to refine structure: *SHELXTL*; molecular graphics: *SHELXTL*; software used to prepare material for publication: *SHELXTL*.

## Supplementary Material

Crystal structure: contains datablocks I, global. DOI: 10.1107/S1600536809039907/xu2606sup1.cif

Structure factors: contains datablocks I. DOI: 10.1107/S1600536809039907/xu2606Isup2.hkl

Additional supplementary materials:  crystallographic information; 3D view; checkCIF report

## Figures and Tables

**Table 1:** Hydrogen-bond geometry (Å, °)

*D*—H⋯*A*	*D*—H	H⋯*A*	*D*⋯*A*	*D*—H⋯*A*
N1—H1⋯O2	0.93 (3)	1.75 (3)	2.671 (3)	173 (3)
N2—H2*A*⋯O1	0.96 (3)	1.87 (3)	2.827 (3)	170 (3)
N2—H2*B*⋯O2^i^	0.91 (4)	1.99 (4)	2.885 (3)	171 (3)
O4—H4*O*⋯O1*W*^ii^	0.89 (3)	1.69 (4)	2.584 (3)	176 (4)
O1*W*—H1*WA*⋯O3^iii^	0.97 (5)	1.91 (5)	2.827 (3)	158 (4)
O1*W*—H1*WB*⋯O1	0.82 (5)	2.14 (4)	2.812 (3)	139 (4)
O1*W*—H1*WB*⋯O3	0.82 (5)	2.31 (5)	3.002 (3)	144 (4)

Symmetry codes: (i) 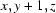; (ii) 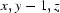; (iii) 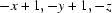.

**Fig. 1.**
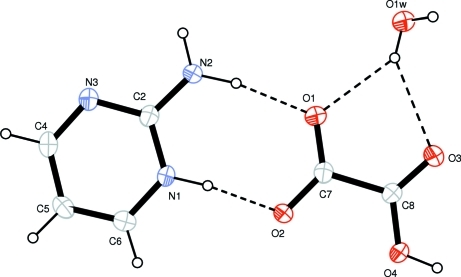
The molecular structure of the title compound, showing the atom-numbering scheme. Displacement ellipsoids are drawn at the 50% probability level. Dashed lines indicate hydrogen bonding.

## supplementary crystallographic information

### Comment

Pyrimidines have been attracted special attention because of unique biological properties, such as fungicides, vermicides, inseticides and medicines (Rabie *et al.*, 2007). Among them, aminopyrimidines are as interesting matters for chemists and pharmacist. They are a part of nucleic bases, cystosine, adenine and guanine which are responsible for molecular recognition and replication of DNA, through the formation and breakage of N—H···N hydrogen bonds (Rospenk & Koll, 2007). 2-Aminopyrimidine (2-apym), with amino group as two H-bond donor atoms and two N atoms as two H-bond acceptors atoms is particularly attractive as a very simple self-complementary prototype for chain formation with other organic molecules. Until now, a lot of co-crystals and proton transfer compounds were synthesized using 2-apym and a variety of carboxylic acid derivatives (Childs *et al.*, 2007). In this report, we choose oxalic acid (OxH_2_) in according to their difference in *p*K_a_.

In the title compound, the oxalic acid is mono-deprotonated while 2-apym is protonated (Fig. 1). The cation is hydrogen bonded to the anion with a cyclic *R*_2_^2^(8) pattern (Table 1). The anionic and cationic moieties link with the water molecule into layers by hydrogen bondings, resulting in beautiful picture as Flag-like strucrure. The O1W–H1WA···O3^iii^ hydrogen bond [symmetry code: (iii) 1 - *x*, 1 - *y*, -*z*] between water and carboxyl group produces the three dimensional supra-molecular structure.

### Experimental

The title compound was synthesized *via* the reaction of OxH_2_ (0.047 g, 0.375 mmol) with 2-apym (0.050 g, 0.5 mmol) in a water solution (5 ml). The solution was stirred for 3 h in 323 K. Colorless crystals were obtained after a week.

### Refinement

Nitrogen- and oxygen-bound H atoms were located in a difference Fourier map and refined isotropically. Carbon-bound H atoms were placed in calculated positions and were refined in riding model with *U*_iso_(H) = 1.2*U*_eq_(C).

### Crystal data

**Table:**

C_4_H_6_N_3_^+^·C_2_HO_4_^−^·H_2_O	*Z* = 2
*M**_r_* = 203.16	*F*(000) = 212
Triclinic, *P*1	*D*_x_ = 1.585 Mg m^−^^3^
Hall symbol: -P 1	Melting point: 300 K
*a* = 6.295 (2) Å	Mo *K*α radiation, λ = 0.71073 Å
*b* = 6.339 (2) Å	Cell parameters from 851 reflections
*c* = 11.111 (4) Å	θ = 3.3–27.7°
α = 75.045 (6)°	µ = 0.14 mm^−^^1^
β = 84.302 (6)°	*T* = 120 K
γ = 86.026 (7)°	Prism, colorless
*V* = 425.8 (2) Å^3^	0.35 × 0.15 × 0.07 mm

### Data collection

**Table:**

Bruker SMART 1000 CCD area-detector diffractometer	1177 reflections with *I* > 2σ(*I*)
Radiation source: normal-focus sealed tube	*R*_int_ = 0.030
graphite	θ_max_ = 27.0°, θ_min_ = 1.9°
φ and ω scans	*h* = −8→8
3983 measured reflections	*k* = −8→8
1835 independent reflections	*l* = −14→14

### Refinement

**Table:**

Refinement on *F*^2^	Primary atom site location: structure-invariant direct methods
Least-squares matrix: full	Secondary atom site location: difference Fourier map
*R*[*F*^2^ > 2σ(*F*^2^)] = 0.055	Hydrogen site location: mixed
*wR*(*F*^2^) = 0.148	H atoms treated by a mixture of independent and constrained refinement
*S* = 1.01	*w* = 1/[σ^2^(*F*_o_^2^) + (0.047*P*)^2^ + 0.72*P*] where *P* = (*F*_o_^2^ + 2*F*_c_^2^)/3
1835 reflections	(Δ/σ)_max_ < 0.001
151 parameters	Δρ_max_ = 0.44 e Å^−^^3^
0 restraints	Δρ_min_ = −0.29 e Å^−^^3^

### Special details

**Table:**

Geometry. All e.s.d.'s (except the e.s.d. in the dihedral angle between two l.s. planes) are estimated using the full covariance matrix. The cell e.s.d.'s are taken into account individually in the estimation of e.s.d.'s in distances, angles and torsion angles; correlations between e.s.d.'s in cell parameters are only used when they are defined by crystal symmetry. An approximate (isotropic) treatment of cell e.s.d.'s is used for estimating e.s.d.'s involving l.s. planes.
Refinement. Refinement of *F*^2^ against ALL reflections. The weighted *R*-factor *wR* and goodness of fit *S* are based on *F*^2^, conventional *R*-factors *R* are based on *F*, with *F* set to zero for negative *F*^2^. The threshold expression of *F*^2^ > σ(*F*^2^) is used only for calculating *R*-factors(gt) *etc*. and is not relevant to the choice of reflections for refinement. *R*-factors based on *F*^2^ are statistically about twice as large as those based on *F*, and *R*- factors based on ALL data will be even larger.

### Fractional atomic coordinates and isotropic or equivalent isotropic displacement parameters (Å^2^)

**Table:**

	*x*	*y*	*z*	*U*_iso_*/*U*_eq_	
N1	0.2235 (4)	0.3419 (4)	0.6301 (2)	0.0225 (5)	
H1	0.235 (5)	0.290 (5)	0.559 (3)	0.037 (9)*	
N2	0.2566 (4)	0.6890 (4)	0.5036 (2)	0.0263 (6)	
H2A	0.270 (5)	0.637 (5)	0.429 (3)	0.027 (8)*	
H2B	0.258 (5)	0.836 (6)	0.492 (3)	0.040 (9)*	
N3	0.2128 (4)	0.6436 (4)	0.7189 (2)	0.0249 (5)	
C2	0.2305 (4)	0.5594 (4)	0.6174 (2)	0.0232 (6)	
C4	0.1869 (4)	0.5030 (5)	0.8289 (3)	0.0264 (6)	
H4A	0.1759	0.5588	0.9010	0.032*	
C5	0.1746 (5)	0.2770 (5)	0.8472 (3)	0.0270 (6)	
H5A	0.1530	0.1818	0.9284	0.032*	
C6	0.1952 (4)	0.2013 (5)	0.7428 (3)	0.0262 (6)	
H6A	0.1895	0.0492	0.7495	0.031*	
O1	0.2756 (3)	0.4892 (3)	0.30262 (17)	0.0276 (5)	
O2	0.2664 (3)	0.1595 (3)	0.43700 (17)	0.0268 (5)	
O3	0.3456 (3)	0.2958 (3)	0.11122 (17)	0.0316 (5)	
O4	0.3088 (4)	−0.0288 (3)	0.24753 (19)	0.0323 (5)	
H4O	0.314 (5)	−0.099 (6)	0.187 (3)	0.039 (9)*	
C7	0.2830 (4)	0.2859 (4)	0.3300 (2)	0.0229 (6)	
C8	0.3161 (4)	0.1829 (4)	0.2173 (2)	0.0230 (6)	
O1W	0.3244 (4)	0.7844 (3)	0.0656 (2)	0.0298 (5)	
H1WA	0.460 (8)	0.772 (7)	0.018 (4)	0.078 (15)*	
H1WB	0.306 (7)	0.661 (8)	0.109 (4)	0.070 (14)*	

### Atomic displacement parameters (Å^2^)

**Table:**

	*U*^11^	*U*^22^	*U*^33^	*U*^12^	*U*^13^	*U*^23^
N1	0.0308 (13)	0.0209 (12)	0.0172 (12)	−0.0009 (9)	−0.0015 (9)	−0.0078 (10)
N2	0.0432 (15)	0.0175 (12)	0.0176 (12)	−0.0027 (10)	−0.0019 (10)	−0.0035 (10)
N3	0.0325 (13)	0.0240 (12)	0.0193 (12)	−0.0007 (10)	−0.0031 (9)	−0.0071 (10)
C2	0.0284 (15)	0.0221 (14)	0.0205 (13)	−0.0005 (11)	−0.0036 (11)	−0.0078 (11)
C4	0.0299 (15)	0.0321 (16)	0.0192 (14)	−0.0013 (12)	−0.0026 (11)	−0.0100 (12)
C5	0.0320 (16)	0.0273 (15)	0.0191 (14)	−0.0006 (12)	−0.0038 (11)	−0.0008 (11)
C6	0.0316 (16)	0.0211 (13)	0.0244 (14)	−0.0023 (11)	−0.0026 (11)	−0.0028 (11)
O1	0.0423 (12)	0.0178 (10)	0.0224 (10)	−0.0008 (8)	−0.0025 (8)	−0.0050 (8)
O2	0.0458 (12)	0.0174 (9)	0.0166 (10)	−0.0010 (8)	−0.0031 (8)	−0.0029 (8)
O3	0.0544 (14)	0.0221 (10)	0.0175 (10)	−0.0041 (9)	−0.0013 (9)	−0.0038 (8)
O4	0.0605 (15)	0.0183 (10)	0.0196 (10)	−0.0015 (9)	−0.0015 (9)	−0.0080 (8)
C7	0.0268 (15)	0.0228 (14)	0.0195 (13)	0.0003 (11)	−0.0016 (11)	−0.0068 (11)
C8	0.0296 (15)	0.0195 (13)	0.0208 (14)	−0.0010 (11)	−0.0022 (11)	−0.0065 (11)
O1W	0.0455 (14)	0.0198 (11)	0.0240 (11)	−0.0019 (9)	−0.0029 (9)	−0.0054 (9)

### Geometric parameters (Å, °)

**Table:**

N1—C6	1.340 (3)	C5—H5A	0.9500
N1—C2	1.353 (3)	C6—H6A	0.9500
N1—H1	0.93 (4)	O1—C7	1.244 (3)
N2—C2	1.320 (3)	O2—C7	1.250 (3)
N2—H2A	0.96 (3)	O3—C8	1.215 (3)
N2—H2B	0.91 (4)	O4—C8	1.299 (3)
N3—C4	1.316 (4)	O4—H4O	0.90 (4)
N3—C2	1.359 (3)	C7—C8	1.545 (4)
C4—C5	1.400 (4)	O1W—H1WA	0.97 (5)
C4—H4A	0.9500	O1W—H1WB	0.82 (5)
C5—C6	1.358 (4)		
			
C6—N1—C2	121.5 (2)	C6—C5—H5A	121.8
C6—N1—H1	119 (2)	C4—C5—H5A	121.8
C2—N1—H1	119 (2)	N1—C6—C5	119.7 (3)
C2—N2—H2A	123.4 (18)	N1—C6—H6A	120.1
C2—N2—H2B	120 (2)	C5—C6—H6A	120.1
H2A—N2—H2B	116 (3)	C8—O4—H4O	119 (2)
C4—N3—C2	116.5 (2)	O1—C7—O2	127.2 (3)
N2—C2—N1	118.4 (2)	O1—C7—C8	115.1 (2)
N2—C2—N3	120.5 (2)	O2—C7—C8	117.7 (2)
N1—C2—N3	121.2 (2)	O3—C8—O4	124.8 (3)
N3—C4—C5	124.7 (3)	O3—C8—C7	121.1 (2)
N3—C4—H4A	117.7	O4—C8—C7	114.1 (2)
C5—C4—H4A	117.7	H1WA—O1W—H1WB	104 (4)
C6—C5—C4	116.4 (3)		
			
C6—N1—C2—N2	−179.3 (3)	C2—N1—C6—C5	−0.5 (4)
C6—N1—C2—N3	1.2 (4)	C4—C5—C6—N1	−0.6 (4)
C4—N3—C2—N2	179.9 (3)	O1—C7—C8—O3	4.3 (4)
C4—N3—C2—N1	−0.6 (4)	O2—C7—C8—O3	−175.8 (3)
C2—N3—C4—C5	−0.7 (4)	O1—C7—C8—O4	−175.5 (2)
N3—C4—C5—C6	1.3 (4)	O2—C7—C8—O4	4.4 (4)

### Hydrogen-bond geometry (Å, °)

**Table:**

*D*—H···*A*	*D*—H	H···*A*	*D*···*A*	*D*—H···*A*
N1—H1···O2	0.93 (3)	1.75 (3)	2.671 (3)	173 (3)
N2—H2A···O1	0.96 (3)	1.87 (3)	2.827 (3)	170 (3)
N2—H2B···O2^i^	0.91 (4)	1.99 (4)	2.885 (3)	171 (3)
O4—H4O···O1W^ii^	0.89 (3)	1.69 (4)	2.584 (3)	176 (4)
O1W—H1WA···O3^iii^	0.97 (5)	1.91 (5)	2.827 (3)	158 (4)
O1W—H1WB···O1	0.82 (5)	2.14 (4)	2.812 (3)	139 (4)
O1W—H1WB···O3	0.82 (5)	2.31 (5)	3.002 (3)	144 (4)

Symmetry codes: (i) *x*, *y*+1, *z*; (ii) *x*, *y*−1, *z*; (iii) −*x*+1, −*y*+1, −*z*.

## References

[bb1] Bruker (1998). *SMART* and *SAINT-Plus* Bruker AXS Inc., Madison, Wisconsin, USA.

[bb2] Childs, S. L., Stahly, G. P. & Park, A. (2007). *Mol. Pharm.* **4**, 323–338. 10.1021/mp0601345 17461597

[bb3] Rabie, U. M., Abou-El-Wafa, M. H. & Mohamed, R. A. (2007). *J. Mol. Struct.* **871**, 6–13.

[bb4] Rospenk, M. & Koll, A. (2007). *J. Mol. Struct.* **844–845**, 232–241.

[bb5] Sheldrick, G. M. (2008). *Acta Cryst.* A**64**, 112–122. 10.1107/S0108767307043930 18156677

